# Expression analysis and functional characterization of the mouse cysteine-rich with EGF-like domains 2

**DOI:** 10.1038/s41598-018-30362-4

**Published:** 2018-08-15

**Authors:** Kentaro Oh-hashi, Keito Fujimura, Junpei Norisada, Yoko Hirata

**Affiliations:** 1 United Graduate School of Drug Discovery and Medical Information Sciences, Gifu University, 1-1 Yanagido, Gifu, 501-1193 Japan; 2 Department of Chemistry and Biomolecular Science, Faculty of Engineering, Gifu University, 1-1 Yanagido, Gifu, 501-1193 Japan

## Abstract

We have previously identified a novel endoplasmic reticulum (ER) stress-inducible protein, namely, cysteine-rich with EGF-like domains 2 (CRELD2), which is predominantly regulated by ATF6. However, few studies on intrinsic CRELD2 have been published. In the present study, we elucidated the expression of intrinsic CRELD2 in mouse tissues and ER stress- treated Neuro2a cells. Among nine tissues we tested, CRELD2 protein in the heart and skeletal muscles was negligible. CRELD2 expression in Neuro2a cells was induced at the late phase after treatment with tunicamycin (Tm) compared with rapid induction of growth arrest and DNA damage inducible gene 153 (GADD153). On the other hand, another ER stress inducer, thapsigargin, increased the intrinsic CRELD2 secretion from Neuro2a cells. We furthermore established CRELD2-deficient Neuro2a cells to evaluate their features. In combination with the NanoLuc complementary reporter system, which was designed to detect protein-protein interaction in living cells, CRELD2 interacted with not only CRELD2 itself but also with ER localizing proteins in Neuro2a cells. Finally, we investigated the responsiveness of CRELD2-deficient cells against Tm-treatment and found that CRELD2 deficiency did not affect the expression of genes triggered by three canonical ER stress sensors but rendered Neuro2a cells vulnerable to Tm-stimulation. Taken together, these findings provide the novel molecular features of CRELD2, and its further characterization would give new insights into understanding the ER homeostasis and ER stress-induced cellular dysfunctions.

## Introduction

Cysteine-rich with EGF-like domains 2 (CRELD2) was first identified as a protein that interacts with human neuronal nicotinic acetylcholine receptor α4 and β2 subunits by the yeast two-hybrid screening and overexpressing study^1^. In addition, CRELD2 was reported to be one of novel androgen receptor target genes in prostate cancer^2^; however, its molecular features have not been fully elucidated.

In the past decade, we first identified this CRELD2 gene as a novel endoplasmic reticulum (ER) stress-inducible gene and have been characterizing it in detail^3^. One of the first findings is that ATF6 positively regulates the transcription of the CRELD2 gene through a well-conserved ER stress response element (ERSE) in its proximal 5′-flanking region. Furthermore, we have evaluated the intracellular traffic and secretion of CRELD2 in cells transiently overexpression of various types of CRELD2 gene under several conditions^4,5^.

The importance of the ER homeostasis has been reported in various types of cells and tissues^6,7^, and the disruption of ER homeostasis, including intracellular calcium and oxidative tone, is known to dampen the folding and modification of newly synthesized transmembrane and secretory proteins within the ER and accumulate abnormal proteins^8^. Similar to an increasing number of downstream targets of the canonical ER resident stress sensors^9,10^, including PKR-like endoplasmic reticulum kinase (PERK)^11^, inositol-requiring enzyme 1 (IRE1)^12^ and activating transcription factor 6 (ATF6)^13^, it has been uncovered that ER homeostasis is regulated by multiple factors in a coordinated manner. Particularly, some ER resident molecular chaperones, such as 78 kDa glucose-regulated protein (GRP78), are predominantly regulated by ATF6 and alleviate the stress by properly folding and degrading unfolded proteins inside the ER lumen and membrane^9,10,13,14^. Considering this scenery, CRELD2 expression might be associated with the relief of ER disturbance under certain adverse circumstances. Hartley *et al*. have reported that CRELD2 interacts with mutant matrilin-3, which is preferentially retained within ER and triggers unfolded protein responses (UPR)^15^; however, precise molecular features of intrinsic CRELD2 have yet to be elucidated. In this study, we first investigated the expression of intrinsic CRELD2 in mouse tissues and Neuro2a cells after treatment with several reagents. Furthermore, we established the CRELD2-deficient Neuro2a cells using a CRISPR/Cas9 system^16,17^ and characterized the molecular features of this protein. Especially, we showed that CRELD2 was not directly associated with typical ER stress-inducible factor expression in response to tunicamycin (Tm), but its depletion rendered the cells vulnerable to Tm stimulation.

## Results

### Expression analysis of intrinsic CRELD2 in mouse tissues and Neuro2a cells

**Figure 1:**
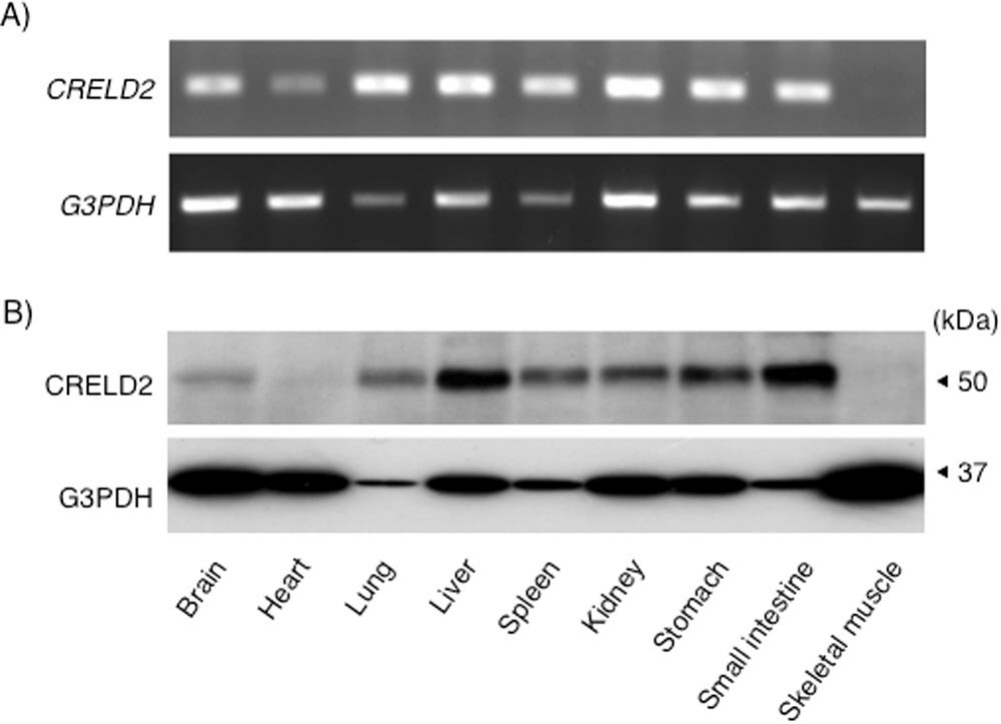
Expression of CRELD2 in mouse tissues. The expression of indicated mRNA (**A**) and protein (**B**) in the mouse tissues was detected by RT-PCR and western blotting analysis as described in the Materials and methods section. Representative data from two (**A**) and three (**B**) mice are shown.

We previously identified CRELD2 as a novel-ER stress inducible gene and reported their features by overexpression of various types CRELD2 gene in cells^3–5,18^. In this study, our original antibody against CRELD2 made it possible to investigate molecular features of intrinsic CRELD2. First, we evaluated the expression of CRELD2 mRNA and protein in nine mouse tissues. As shown Fig. 1A, CRELD2 mRNA was detected in all tissues except for the skeletal muscle. CRELD2 protein was detected at approximately 50 kDa (Fig. 1B), which is almost accorded with the predicted molecular size and CRELD2-overexpressing study^18^. CRELD2 protein expression in the heart and skeletal muscle was negligible.

**Figure 2:**
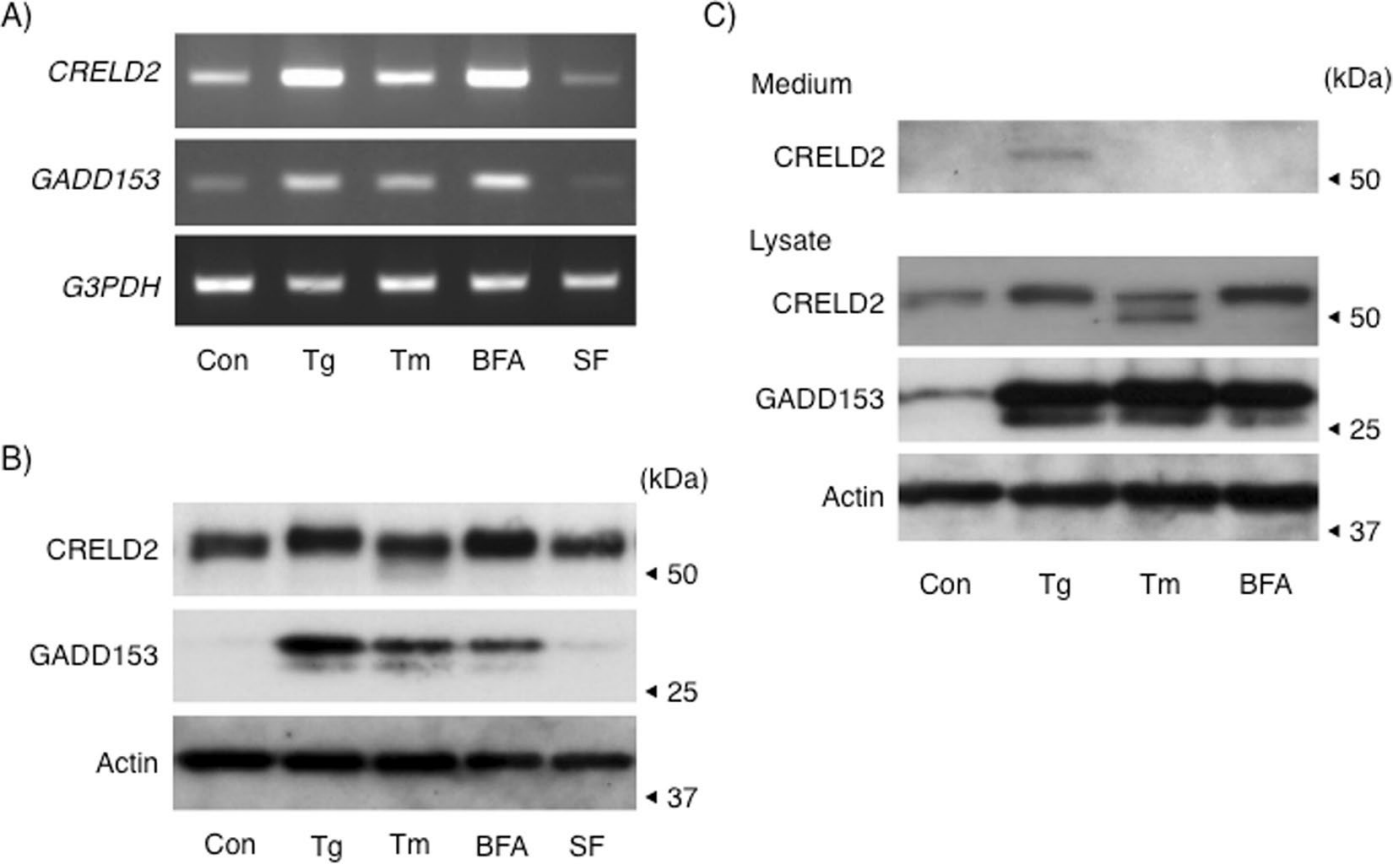
Expression of CRELD2 in Neuro2a cells. Neuro2a cells were treated with or without thapsigargin (Tg, 0.1 μM), tunicamycin (Tm, 1 μg/ml), breferdin A (BFA, 2.5 μg/ml) or serum-deprived medium (serum free, SF) for 8 h (**A**) and 12 h (**B**). (**C**) Neuro2a cells under the serum-deprived medium were treated with Tg, Tm, BFA or vehicle for 12 h. After treatment, secreted CRELD2 (medium) and the indicated proteins in each cell lysate (lysate) were prepared as described in the Materials and methods section. The expression of the indicated mRNA (**A**) and protein (**B**,**C**) was detected by RT-PCR and western blotting analysis as described in the Materials and methods section. Representative data from more than three independent experiments are shown.

Among the tissues we tested, CRELD2 expression in brain was not so high. On the other hand, we first identified CRELD2 as a novel ER stress-inducible gene using microarray analysis of Tg-treated mouse neuroblastoma cell-line, Neuro2a^3^, and have been employing this Neuro2a cells to elucidate several ER stress gene expression (e.g., MANF, Chac1 and GADD153) in detail^17,19,20^. On the basis of our previous findings, we next investigated the expression of CRELD2 mRNA and protein in Neuro2a cells after treatment with three well-used ER stress-inducing stimuli (Tg, Tm and BFA). In line with our previous study, treatment with three reagents but not serum starvation (serum free, SF) induced both CRELD2 and GADD153 mRNA expression in Neuro2a cells (Fig. 2A). CRELD2 protein was already expressed without stimuli and its expression was hardly elevated by 12 h-treatment (Fig. 2B). However, GADD153 protein was remarkably induced by Tg, Tm and BFA. A small portion of CRELD2 protein with a lower molecular size was detected after Tm-treatment. Since we previously showed that treatment with EndoH glycosidase decreased a molecular size of intracellular CRELD2^4^, CRELD2 protein with a lower molecular size was considered as a de-glycosylated form. On the other hand, Tg-treatment only slightly shifted the entire band of CRELD2 upward.

**Figure 3:**
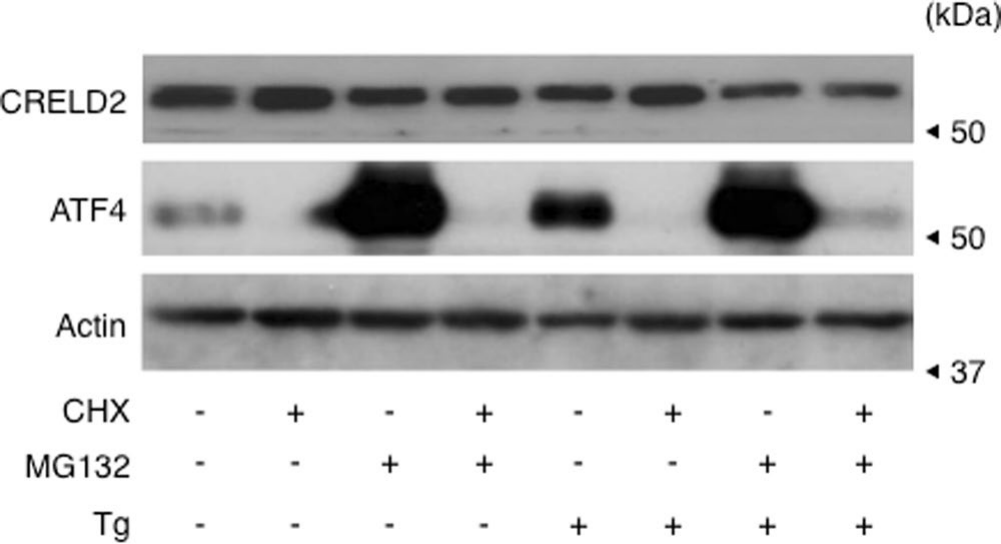
Evaluation of stability of intrinsic CRELD2 protein in Neuro2a cells. Neuro2a cells were treated with Tg (0.1 μM), cycloheximide (CHX, 10 μg/ml), MG132 (10 μM) or vehicle for 6 h, and expression of the indicated protein was detected as described in the Materials and methods section, and reproducibility was confirmed.

To understand this discrepancy between CRELD2 mRNA and protein expression, we then tried to detect the intrinsically secreted CRELD2 from Neuro2a cells cultured under serum-deprived condition since we reported that CRELD2 is a secretory factor through the ER – Golgi pathway using the transient overexpression model^4^. Interestingly, CRELD2 protein in the serum free medium was detected 12 h after Tg-treatment; however, secreted CRELD2 under other conditions was negligible (Fig. 2C). Next, we examined stability of CRELD2 protein in Neuro2a cells by treatment with cycloheximide (a protein synthesis inhibitor), and/or MG132 (a proteasome inhibitor) together with Tg for 6 h. As shown in Fig. 3, ATF4, the well-known ER stress-inducible transcription factor and a substrate for proteasome, was stabilized with MG132, and its expression was abolished by CHX; however, the amount of CRELD2 protein was hardly affected by each treatment.

**Figure 4:**
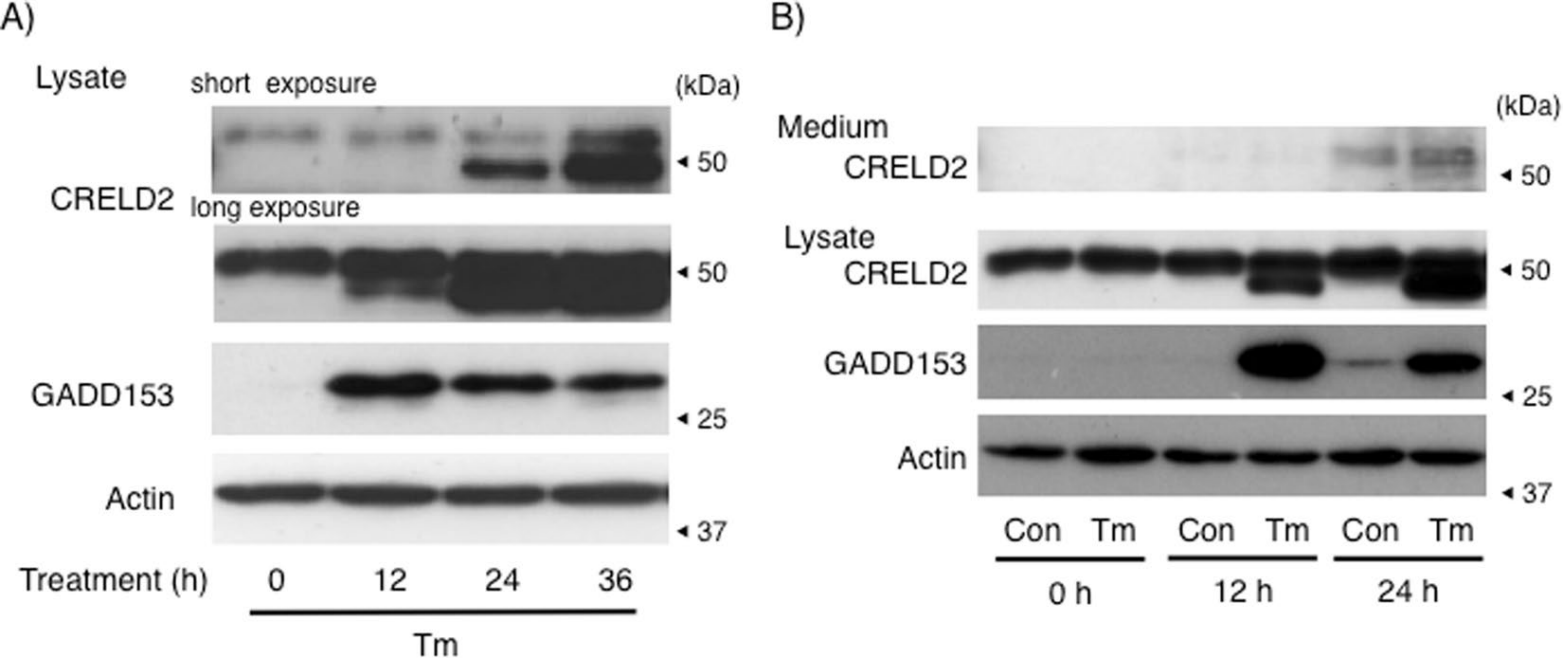
Long term treatment with tunicamycin induced CRELD2 protein in Neuro2a cells. Neuro2a cells in serum-containing culture medium (**A**) or serum-deprived medium (**B**) were treated with tunicamycin (Tm, 1 μg/ml) for the indicated time. Expression of the indicated protein in each culture medium and cell lysate was detected as described in the Materials and methods section, and reproducibility was confirmed.

We then investigated the effect of long-term treatment with Tm on the CRELD2 expression in Neuro2a cells in the presence (Fig. 4A) or absence (Fig. 4B) of serum. In line with the above study, 12 h of treatment with Tm partly shifted the band of CRELD2 downward, and further treatment with Tm markedly increased both the glycosylated and the de-glycosylated forms. Only weak signal of secreted CRELD2 was detected 24 h after the incubation of cells under serum-free medium (Fig. 4B).

**Figure 5:**
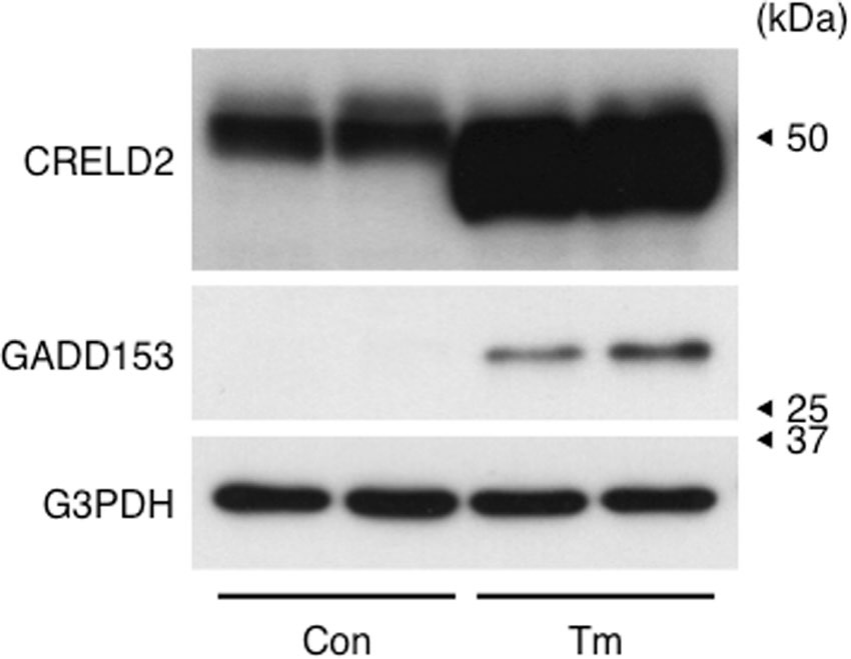
Intraperitoneal administration of tunicamycin induced CRELD2 protein in mouse liver. Twenty-four hours after intraperitoneal injection of Tm (1 mg/kg) (n = 4) or vehicle (n = 4) into ddY mice, each liver was isolated and expression of the indicated protein was detected as described in the Materials and methods section.

Next, we investigated whether Tm-administration induced CRELD2 protein *in vivo* 24 h after intraperitoneal injection of Tm into mouse. In consistent with induction of CRELD2 protein in Tm-treated Neuro2a cells, Tm-administration dramatically induced CRELD2 protein in mouse liver (Fig. 5). Especially, increase in unglycosylated CRELD2 was prominent.

### Establishment and characterization of CRELD2-deficient cells

**Figure 6:**
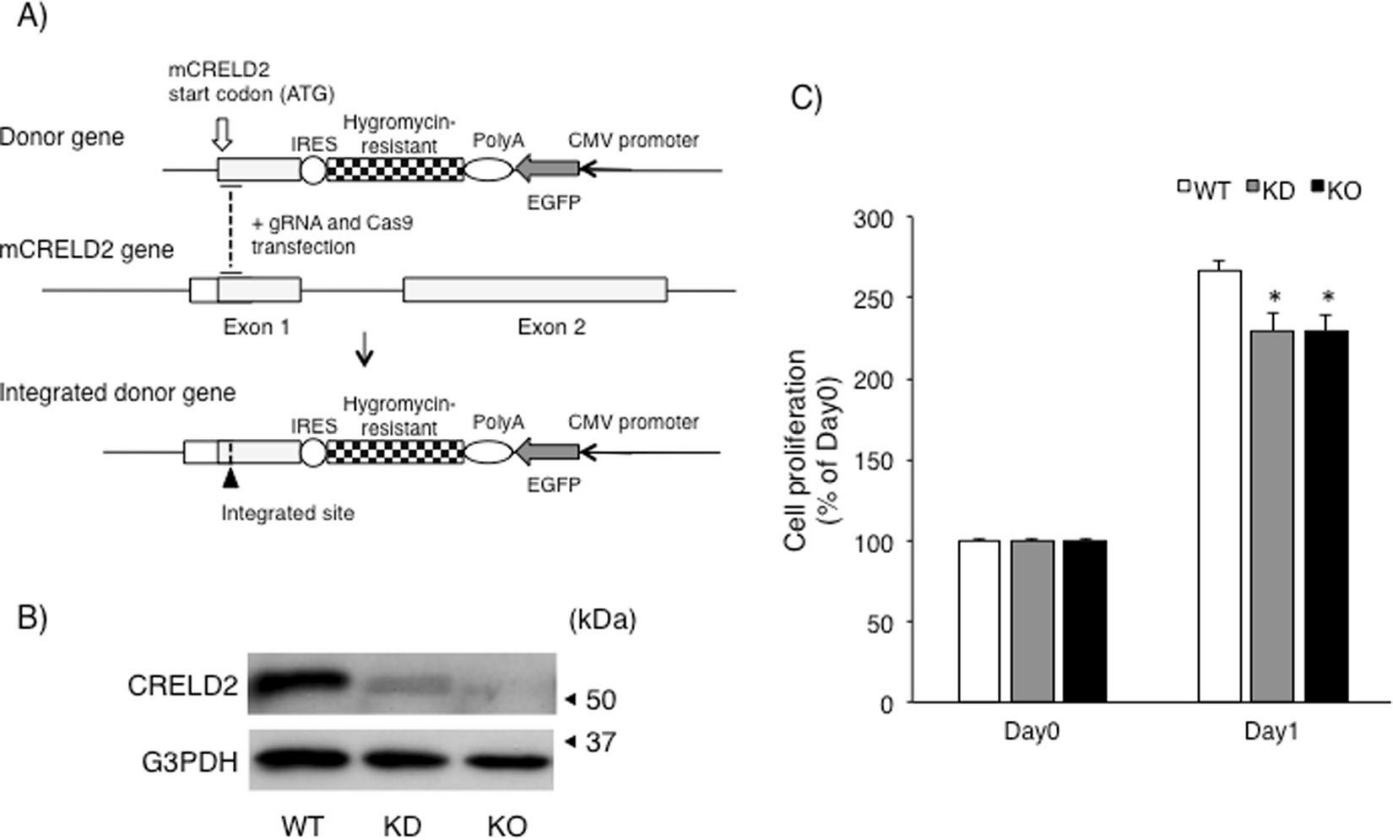
Establishment of CRELD2 deficient Neuro2a cells using CRISPR/Cas9 system. (**A**) The schematic structure of the donor gene, CRELD2-IRES-hygro pGL, and the strategy for establishing the CRELD2-deficient cells. The open arrow and arrowhead indicate the translation start site of mouse CRELD2 and the integrated site, respectively. (**B**) Expression of the indicated protein in parental wild-type (WT), hygromycin selected (KD) and a cloned (KO) Neuro2a cells was detected as described in the Materials and methods section. (**C**) The parental (WT), hygromycin selected (KD) and a cloned (KO) Neuro2a cells were cultured in 96-well plates for the indicated days, and cell proliferation was measured as described in the Materials and methods section. Each value represents the mean ± SEM from more than 5 independent cultures.

**Figure 7:**
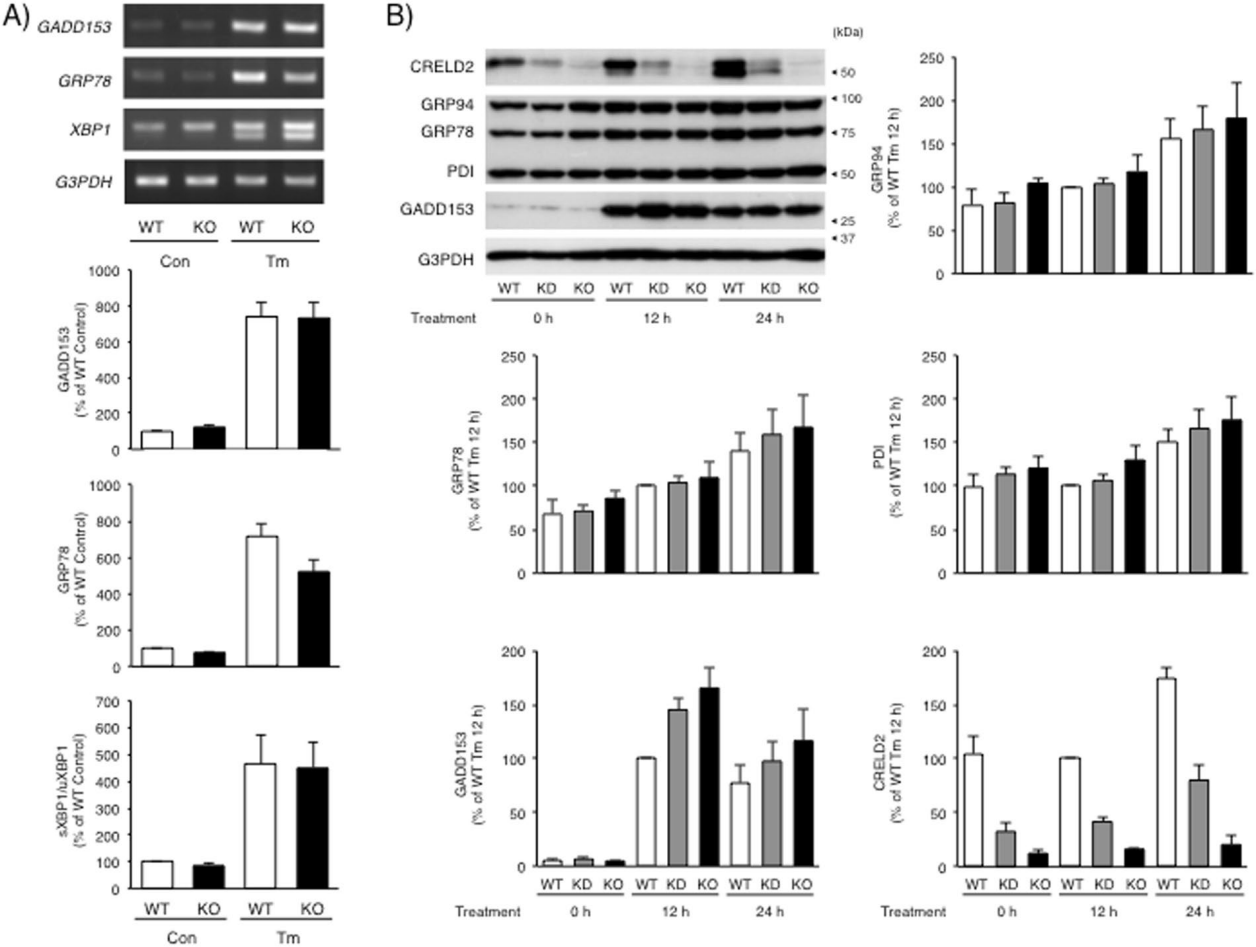
Expression of ER stress-responsive factors in response to Tm in the CRELD2-deficient Neuro2a cells. (**A**) The parental or cloned CRELD2-deficient (CRELD2 KO) Neuro2a cells were treated with Tm (1 μg/ml) or vehicle for 12 h and expression level of each mRNA was determined as described in the Materials and methods section. (**B**) The parental, hygromycin selected (KD) and a cloned (KO) Neuro2a cells were treated with Tm for the indicated time and the expression level of each protein was determined as described in the Materials and methods section. Relative amount of genes and proteins was calculated as described in the Materials and methods section. The values obtained from untreated parental cells (**A**) or the parental Neuro2a cells after 12 h of treatment with Tm (**B**) were considered as “100%”. Each value represents the mean ± SEM from 5 independent cultures.

We next established the CRELD2-deficient Neruo2a cells based on our recent study^17^ (Fig. 6A,B). Since cells expressed only small amount of CRELD2 after selection with hygromycin (CRELD2 knock-down cells, KD), we further cloned the cells lacking the intrinsic CRELD2 protein (CRELD2 knock-out cells, KO) (Fig. 6B) and used both CRELD2-deficient cells (KD and KO) for the following experiments. As shown in Fig. 6C, both CRELD2-deficient cells showed a tendency to proliferate slowly. We further evaluated the expression of typical ER stress-inducible factors which are downstream of ATF6, PERK and IRE1, respectively. As shown in Fig. 7A,B, expression of each mRNA and protein did not significantly change among each cell. In consistent with previous study on Tm-treated Neuro2a cells^17^, protein expression of GADD153, one of pro-apoptotic factors under several pathological conditions, remarkably increased 12 h after Tm-treatment and slightly declined at 24 h. The amount of GADD153 protein in both CRELD2 deficient cells after Tm-treatment was slightly higher in comparison with that in the parental Neuro2a cells; however, the difference was not statistically significant.

**Figure 8:**
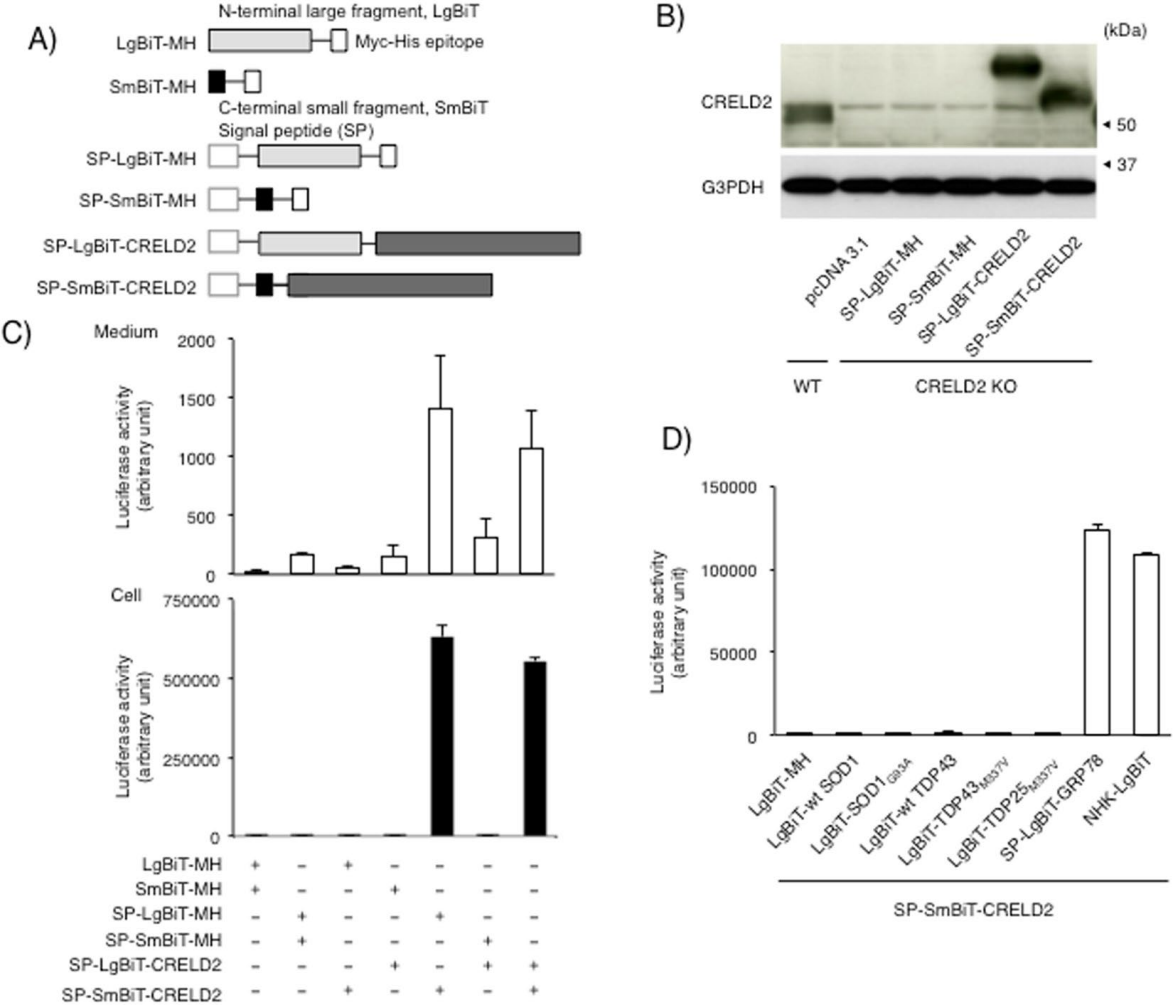
Studies on the intracellular behavior of CRELD2 protein in living Neuro2a cells using a NanoLuc complementary reporter assay. (**A**) Schematic structures of NanoBiT-tagged constructs used in this study. (**B**) After transfection of the indicated CRELD2 gene into the cloned CRELD2-deficient cells, expression of the indicated protein was determined as described in the Materials and methods section. (**C**) Twenty-hour hours after transfection of the indicated genes into the cloned CRELD2 deficient cells, culture medium was replaced with OPTI-MEM and cells were cultured for additional 6 h. After incubation, culture medium was collected for measurement of extracellular NanoBiT activity (open bars). For measurement of intracellular NanoBiT activity, culture medium was replaced with fresh OPTI-MEM, and the diluted substrate was directly added to each well to measure the NanoBiT activity (filled bars). (**D**) Thirty hours after transfection of SP-SmBiT-CRELD2 together with the indicated gene, culture medium was replaced with fresh OPTI-MEM and the diluted substrate was directly added to each well to measure the intracellular NanoBiT activity in living cells. Each value represents the mean ± SEM from 3 independent cultures.

Since it is predicted that CRELD2 possesses PDI-like activity through interaction with several proteins such as matrillin-3, laminin-5 β3, collagen VI and thrombospondin-1^15^, we prepared several constructs for the NanoLuc complementary reporter system (NanoBiT)  to investigate whether protein-interaction with CRELD2 occurred in living cells^21,22^. To avoid the effect of intrinsic CRELD2 on this NanoBiT assay, we employed CRELD2 deficient cells (KO) established in this study. As shown in Fig. 8A–C, high NanoLuc activity through interaction between LgBiT- and SmBiT-epitope tagged proteins was observed in SP-LgBiT-CRELD2/SP-SmBiT-CRELD2 expressing cells. Interestingly, similar activity was observed in cells expressing SP-LgBiT-MH and SP-SmBiT-CRELD2 (Fig. 8C). Since cells expressing LgBiT-MH/SP-SmBiT-CRELD2 did not show a higher NanoBiT activity, we investigated whether CRELD2 preferentially interacted with proteins within the ER-Golgi pathway. Among proteins we tested, SP-LgBiT-GRP78 and a null Hong Kong variant of α-1-antitrypsin (NHK)^23^ having LgBiT at the C-terminus showed apparent NanoBiT activity; however, transfection of SP-SmBiT-CRELD2 together with the cytosolic or nuclear localizing proteins, namely, SOD1 and TDP43^24^, did not show any NanoBiT activity in living cells (Fig. 8D). As well, cells expressing SP-LgBiT-CRELD2 together with SP-SmBiT-GRP78 or NHK-SmBiT into this CRELD2 deficient cells also showed a higherNanoBiT activity (data not shown).

**Figure 9:**
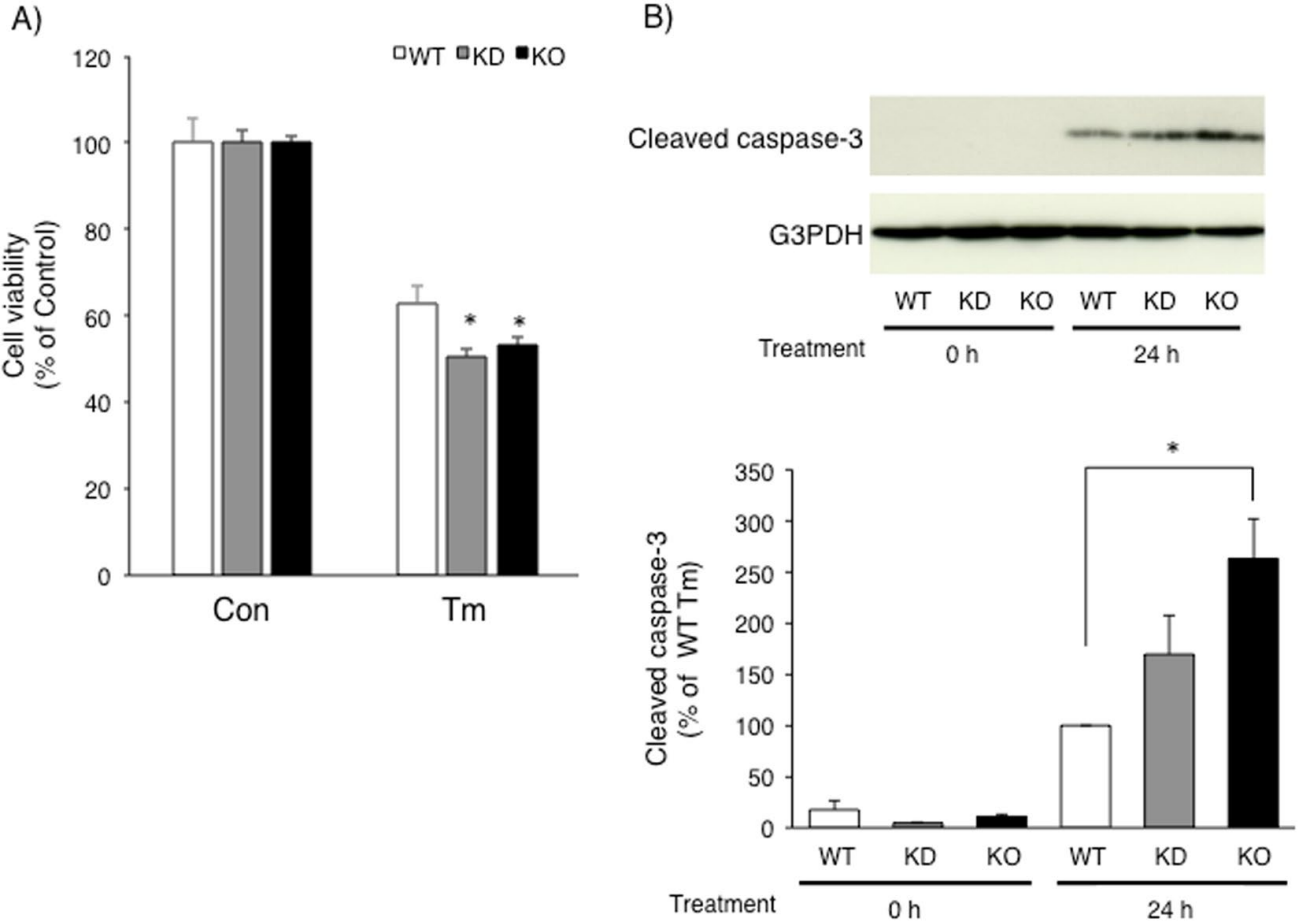
CRELD2 deficiency in Neuro2a cells caused the cells vulnerable to tunicamycin. (**A**) The parental wild-type (WT), hygromycin selected (KD) and a cloned (KO) Neuro2a cells in 96-well plate were treated with Tm (1 μg/ml) or vehicle (Control, Con) for 24 h. Cell viability was measured as described in the Materials and methods section. Each value represented the mean ± SEM from 5 independent cultures. (**B**) The parental (WT), hygromycin selected (KD) and a cloned (KO) Neuro2a cells were treated with Tm for 24 h. The expression levels of the indicated protein were determined as described in the Materials and methods section. The values obtained from the parental Neuro2a cells after 24 h of treatment with Tm were considered as 100%. Relative amount of cleaved caspase-3 was calculated as described in the Materials and methods section. Each value represents  the mean ± SEM from 5 independent cultures.

Finally, we investigated whether CRELD2 deficiency influenced Neuro2a cell death in response to Tm treatment. As shown in Fig. 9A, cell viability based on WST-1 assay slightly but significantly decreased in both CRELD2 deficient cells after 24 h of treatment with Tm. In parallel, expression of the cleaved caspase-3 expression levels 24 h after Tm treatment was elevated in CRELD2 deficient cells; especially, cleaved caspase-3 expression levels in CRELD2 KO cells were significantly higher than in the parental Neuro2a cells (Fig. 9B).

## Discussion

CRELD2 was first identified as a factor regulating the intracellular trafficking of acetylcholine receptor α4 and β2 subunits^1^. Then, CRELD2 mRNA expression was documented in several types of cells and tissues including cancerous cells^1–3,25–27^; however, CRELD2 protein expression has been poorly elucidated. We prepared an original antibody that specifically recognizes intrinsic CRELD2 protein and detected it in adult mouse tissues and Neuro2a cells after ER stress-inducing stimuli. Among the nine mouse tissues we tested, CRELD2 protein in the heart and skeletal muscles was negligible. Considering that almost all ER stress-inducible factors associating protein-folding, -modification, -degradation or -transport are basically and ubiquitously expressed, it is thought that CRELD2 might be a component regulating a certain ER homeostasis. In Neuro2a cells, CRELD2 protein was hardly elevated after 12 h of treatment with Tm, even though GADD153 was dramatically induced. Both CRELD2 and GADD153 genes have an ERSE element in each 5′-flanking region and both mRNAs were actually induced in response to ER stress inducers. It is therefore considered that post-transcriptional regulations might differ between CRELD2 and GADD153 protein expression. Similarly, GRP78 and 94 kDa glucose-regulated protein (GRP94), the well-known ATF6-dependent chaperones^9^, were gradually induced from 12 to 24 h. Interestingly, CRELD2 protein, especially an unglycosylated form, in mouse liver was dramatically induced 24 h after Tm-administration as observed in Neuro2a cells. It is therefore thought that mechanisms of Tm-induced CRELD2 expression are conserved among several cells and tissues.

On the other hand, we observed that intracellular calcium mobilization by Tg-treatment induced the intrinsic CRELD2 secretion, suggesting that secretion of intrinsic CRELD2 is dependent on the intracellular calcium ion as well as our CRELD2-overexpressing study^5^. This finding indicates that little increase in intracellular CRELD2 protein under this Tg-treatment might be due to its increased secretion. Since it has recently been reported that elevation of urinary CRELD2 in ER stress-associated renal disease would be a potential biomarker^28^, it is thought that CRELD2 is secreted from several types of cells in varying degree, and they might influence certain cellular behaviors. On the other hand, our findings implicate that a large amount of CRELD2 is localized inside the cells. In this study, we prepared our original antibody against CRELD2 and could evaluate intrinsic CRELD2 protein in mouse tissues and Neuro2a cells; however, this was not sensitive enough to detect CRELD2 in body fluids including blood. Therefore, establishment of more sensitive assay for CRELD2 is required to uncover its humoral features as a biomarker and/or an intercellular signaling factor.

Since BFA dampens ER-Golgi transport by disrupting Golgi structure, we expected the accumulation of CRELD2 protein inside the cells. However, BFA hardly increased the amounts of intra- and extracellular CRELD2 under the current condition. To understand this issue, we investigated the stability of CRELD2 protein with or without ER stress because even IRE1^12^ and ATF6^13^, major ER stress transducers, are substrates for ER-associated degradation (ERAD)^29,30^. Unexpectedly, inhibitors of protein synthesis and proteasome hardly fluctuated CRELD2 expression, indicating that CRELD2 protein is relatively stable inside the cells. We therefore cannot explain this discrepancy between CRELD2 mRNA and protein expression in BFA-treated cells well; however, we and other groups have reported that BFA triggers not only ER stress but also Golgi stress^31,32^. This distinct pharmacological action might influence the behavior of intracellular CRELD2 protein though the precise mechanisms and signaling pathways of Golgi stress have not been fully characterized.

Regarding functions of CRELD2, Zhang *et al*. have reported that CRELD2 induces the osteogenic differentiation of mesenchymal stem cells^33^. We previously reported that CRELD2 secretion was significantly enhanced by co-transfection of MANF, another ER stress-inducible trophic factor^18,19,34–36^, with CRELD2, and its positive action was dependent on each C-terminal motif (REDL and RTDL)^18,36^; however, it is unclear whether CRELD2 acts as a humoral factor. Recent studies have found that specific induction of certain gene subsets in response to a non-pathological mild ER stress gets involved in osteogenesis, chondrogenesis and glial differentiation^37–39^. Therefore, adequate amount of intracellular CRELD2 protein under resting condition might participate in the processing and/or transport of certain secretory factors and transmembrane proteins from the ER to the Golgi apparatus. Actually, this novel NanoLuc complementary reporter assay by transfection of NanoBiT-tagged CRELD2 together with several types of proteins suggests that CRELD2 is associated with multiple ER localizing proteins, although it is unclear whether their interaction is constitutive or transient and how strongly they interact within living cells. Since we transfected NanoBiT-tagged CRELD2 into CRELD2-deficient cells, it is thought that this model was relatively close to the intrinsic status of CRELD2. Considering that this NanoBiT assay is required for the correct orientation and conformation between the two proteins, detected NanoBiT activities through interaction of CRELD2 with the tested proteins imply that CRELD2 interacts with multiple ER-localizing proteins similar to chaperones within the crowded ER and controls the protein homeostasis through its putative PDI-like activity^15^. Among the proteins showing the NanoBiT activity, the values from cells expressing SP-LgBiT-MH/SP-SmBiT-CRELD2 and SP-LgBiT-CRELD2/SP-SmBiT-CRELD2, respectively were approximately 10 times higher than those from other cells. It is thought that the former shows less steric hinderance between SP-LgBiT-MH and SP-SmBiT-CRELD2 to reconstitute proper NanoLuc activity. The latter implies that a part of CRELD2 protein forms homo-dimer and/or -oligomer both inside and outside of the cells. Though further molecular characterization is required to uncover the precise function, our CRELD2 deficient cells partly showed the role of CRELD2 in cellular homeostasis within the ER. On the other hand, CRELD2 was not associated with the activation of three canonical sensors, namely, ATF6, PERK and IRE1^11–13^, since the downstream gene expression of each sensor was hardly affected by CRELD2 deficiency. Under current conditions, CRELD2 was not indispensable for cell survival; however, CRELD2 deficient cells tends to get out of order since cell proliferation and cell viability after Tm treatment in the CRELD2-deficient cells was respectively attenuated. On the other hand, amount of GADD153 protein, a well-known pro-apoptotic factor^40^, in the CRELD2-deficient cells after 12 h- and 24 h-treatment was slightly high but not statistically significantly. Since not only GADD153 but also activation of multiple factors (e.g., Bim and/or Bnip3) leads to cell death under ER stressed condition^41,42^, further characterization of CRELD2 inside and outside cells is required to understand the mechanisms that CRELD2 deficiency increases the vulnerability to Tm stimulation in Neuro2a cells.

In conclusion, we comprehensively analyzed intrinsic CRELD2 expression in mouse tissues and Neuro2a cells for the first time and showed its ubiquitous expression and secretory feature. Moreover, we first established CRELD2 deficient cells using CRISPR/Cas9 system, showing that CRELD2 is not essential for cell survival and ER stress responsiveness; however, CRELD2 deficiency renders cells vulnerable to certain stresses. Our combinational study with the NanoBiT assay implies a certain role of CRELD2 in controlling proteins within ER. Further characterization of CRELD2 inside and outside cells under pathophysiological conditions may give new insights into diagnosis, prevention and treatments of the ER stress-related diseases.

## Materials and Methods

### Construction of plasmids

Each construct for the NanoLuc complementary reporter system, called NanoBiT (NB) assay, was prepared as previously described^21,22^. In brief, N-terminal large (LgBiT) and C-terminal small (SmBiT) fragments derived from NanoLuc (Promega, USA) were subcloned into pcDNA3.1 Myc/his (MH) vector^22^. A signal peptide sequence (SP) derived from mouse MANF gene was added to LgBiT-MH and SmBiT-MH at their N-terminus, SP-LgBiT-MH and SP-Sm-BiT-MH. Human SOD1 (wild-type (wt) and G93A), human TDP43 (wt and M337V) and TDP25_M337V_ having LgBiT or SmBiT at the N-terminus were subcloned into pcDNA3.1 vector as previously described^22^. A null Hong Kong variant gene of α-1-antitrypsin (NHK)^23^ with each NanoBiT fragment at the C-terminus was cloned into pcDNA3.1. For mouse CRELD2 and GRP78 genes^4^, LgBiT or SmBiT epitope was inserted downstream of each signal peptide sequence (SP) and then subcloned into pcDNA3.1 vector to make SP-LgBiT-CRELD2, SP-SmBiT-CRELD2, SP-LgBiT-GRP78 and SP-SmBiT-GRP78. gRNAs against mouse CRELD2 (5′-GCTGCTGCTGCTGCCGCCGCC-3′) aligned with tracer RNA were inserted into a pcDNA3.1-derived vector with a U6 promoter. To prepare a donor gene, a DNA fragment coding the N-terminal region of mouse CRELD2 (123 bp from the translation start site) was fused with hygromycin-resistant gene via IRES and inserted into a pGL3-derived vector^17^. The hCas9 construct (#41815) used in this study was obtained from Addgene^16^.

### Cell culture and treatment

Neuro2a cells were maintained in Dulbecco’s Modified Eagle’s Minimum Essential Medium containing 5% fetal bovine serum. Transfection of the indicated constructs was performed using the PEI-MAX reagent (Polysciences) as previously described^5,17^. For the establishment of CRELD2-deficient cells, Neuro2a cells transfected with the indicated constructs; the gRNA, hCas9 and donor genes, were cultured with hygromycin, and the resultant cells were used in this study^17^. During these selections, the parental wild-type (WT) Neuro2a cells were maintained with the normal culture medium and were used as control cells for the following experiments. A CRELD2-deficient single clone was obtained after the seeding and growth of a cell in 96-well plate. In each experiment, parental and each deficient cell were seeded into 96- or 12-well plates or 3.5-cm dishes with non–hygromycin containing culture medium. After that, the cells were treated with or without thapsigargin (Tg, 0.1 μM), tunicamycin (Tm, 1 μg/ml), breferdin A (BFA, 2.5 μg/ml), cycloheximide (CHX, 10 μg/ml) (Sigma-Aldrich), MG132 (MG, 10 μM) (Peptide Institute) or serum-deprived medium (serum free, SF) for the indicated time period.

### Mice

ddY mice (4–6 wk male) were purchased from SLC (Japan). The usage of mice was approved by the Animal Care and Use Committee of Gifu University and the mice were treated in accordance with the Regulations of Animal Experiments in Gifu University. Twenty-four hours after intraperitoneal injection of Tm (1 mg/kg) or vehicle, each liver was isolated and used for western blotting analysis.

### Measurement of cell proliferation and viability

For the measurement of cell proliferation using Cell Counting Kit (Dojindo)^17^, the same numbers of parental or CRELD2-deficient Neuro2a cells in a 96-well plate were cultured with the normal culture medium for the indicated days with or without Tm-treatment. During the last hour, WST-1 solution was added to each well and incubated at 37 °C according to the manufacturer’s instruction. The difference between absorbance at 450 nm and 620 nm was measured as an indicator of cell proliferation and viability. Each absorbance in the parental and CRELD2-deficient cells at day 0 or without Tm-treatment was defined as 100%, respectively.

### Reverse transcription polymerase chain reaction (RT-PCR)

To estimate the expression level of each gene by RT-PCR, total RNA was extracted from cells and indicated mouse tissues (6 wk male ddY mice) (SLC, Japan) with TRI Reagent, an equal amount of total RNA from each sample was converted to cDNA by reverse transcription using random nine-mers to prime SuperScript III Reverse Transcriptase (RT) (Life Technologies) as previously described^17,23^. Each cDNA was added to a PCR reaction mixture for amplification (Taq PCR kit, Takara). The PCR primers used in this study are as follows: CRELD2 sense primer 5′-AGAGGAACGAGACCCACAGCA-3′, CRELD2 antisense primer 5′-TGTGCACTGTCCACTCTCCTTGGT-3′, GADD153 sense primer 5′-GAATAACAGCCGGAACCTGA-3′, GADD153 antisense primer 5′-GGACGCAGGGTCAAGAGTAG-3′; glyceraldehyde 3-phosphate dehydrogenase (G3PDH) sense primer, 5′-ACCACAGTCCATGCCATCAC-3′, G3PDH antisense primer, 5′-TCCACCACCCTGTTGCTGTA-3′; GRP78 sense primer 5′-ACCAATGACCAAAACCGCCT-3′, GRP78 antisense primer 5′-GAGTTTGCTGATAATTGGCTGAAC-3′; XBP1 sense primer, 5′-ACGCTTGGGAATGGACACG-3′, XBP1 antisense primer and 5′-ACTTGTCCAGAATGCCCAAAAG-3′.

The typical reaction cycling conditions were 30 sec at 96 °C, 30 sec at 58 °C and 30 sec at 72 °C. The results represented 20–30 cycles of amplification. The products were separated by electrophoresis on 2.0% agarose gels and visualized using ethidium bromide. The expression level of each gene was analyzed using the ImageJ software (National Institutes of Health) and normalized by the values obtained from parental Neuro2a cells without treatment.

### Western blotting analysis

We detected the amount of each protein in the cell lysate from cells and ddy mouse tissues as previously described^5,17^. The cells were lysed and sonicated with homogenization buffer (20 mM Tris-HCl (pH 8.0) containing 137 mM NaCl, 2 mM EDTA, 10% glycerol, 1% TritonX-100, 1 mM PMSF, 10 μg/ml leupeptin and 10 μg/ml pepstatin A). For the preparation of mouse tissue lysates, each tissue was homogenized with the above buffer, and they were briefly centrifuged. Each collected supernatant was used for the following experiments. After the protein concentration was determined using Bradford protein assay dye reagent (BioRad), each cell lysate was dissolved in an equal amount of 2 × sodium dodecyl sulfate (SDS)-Laemmli sample buffer (62.5 mM Tris-HCl (pH 6.8), 2% SDS, 10% glycerol and 12% 2-mercaptoethanol (2-ME)). To detect CRELD2 in the culture medium, equal amounts of each culture medium were resuspended in SDS-Laemmli sample buffer. Equal amounts of each sample from lysates and culture medium were separated on 10 or 12.5% SDS-polyacrylamide gels, transferred onto polyvinylidene difluoride membranes (GE Healthcare) and identified by enhanced chemiluminescence (GE Healthcare) using antibodies against cleaved caspase-3 (Cell Signaling Technology), GADD153 (Santa Cruz Biotechnology), G3PDH (Acris) and KDEL proteins (MBL). The rabbit polyclonal anti-CRELD2 antibody was prepared against 17 aa synthetic peptides (49–65 aa in mouse CRELD2) (Sigma). The expression level of each protein was analyzed using the ImageJ software (National Institutes of Health), and the relative amount of each protein was calculated based on the G3PDH value obtained from the identical lysate^17^. The protein expression of each lysate was normalized to the values obtained from the parental Neuro2a cells as described in figure legends.

### Measurement of NanoBiT activity in living cells

After transiently overexpressing the indicated constructs and culturing cells for the indicated time, each culture medium was replaced with fresh OPTI-MEM (100 μl), and the cells were cultured for the indicated time. After that, 50 μl of culture medium was collected and remaining medium was replaced with fresh equal volume of OPTI-MEM. The diluted NanoLuc substrate for living cells (Promega) was added to each well, and extra- and intracellular luciferase activities were measured by Luminescencer-JNR II (ATTO).

### Statistical analysis

The results are expressed as the means ± SEM. Statistical analyses were carried out using one-way ANOVA followed by Tukey’s test. *p* < 0.05 was considered statistically significant.
